# CAF-Targeting Antibody–Drug Conjugates (ADCs) in Solid Cancers

**DOI:** 10.3390/cancers17101654

**Published:** 2025-05-14

**Authors:** Kostas A. Papavassiliou, Amalia A. Sofianidi, Athanasios G. Papavassiliou

**Affiliations:** 1 First University Department of Respiratory Medicine, ‘Sotiria’ Chest Hospital, Medical School, National and Kapodistrian University of Athens, 11527 Athens, Greece; konpapav@med.uoa.gr; 2 Department of Biological Chemistry, Medical School, National and Kapodistrian University of Athens, 11527 Athens, Greece; amaliasof@med.uoa.gr

The tumor microenvironment (TME) constitutes a major part of solid malignancies and within it, cancer-associated fibroblasts (CAFs) continuously interact with cancer cells, fostering their growth and survival. Notably, CAFs create a fibrotic sheath, impeding the penetration of anticancer agents in the TME. Targeting CAFs is a promising yet challenging therapeutic strategy against various types of solid cancer. Nevertheless, with the advent of antibody–drug conjugates (ADCs), we may successfully eliminate tumor-promoting CAFs and inhibit tumor growth. Several new studies have developed CAF-targeting ADCs that appear to be effective in suppressing cancer, suggesting that this therapeutic approach is feasible and beneficial. As a proof of concept, there are already a few preclinical and clinical trials underway evaluating CAF-targeting ADCs in different solid malignancies.

ADCs targeting fibroblast activation protein (FAP), a type II integral membrane protease well known for its stable and durable expression on the surface of CAFs [1], have already been developed. FAP is negligibly expressed in normal tissues, primarily involved in the process of tissue remodeling and wound healing; rather, its expression is limited to cancerous tissues during adulthood [2]. Targeting FAP is an attractive strategy that could be implemented in the treatment of various malignancies, as FAP is expressed in the stroma of every solid tumor [2]. Intriguingly, FAP expression has been associated with dismal clinical outcomes [3,4], and in this regard, research has recently developed an ADC, labeled huB12-monomethyl auristatin E (MMAE; a synthetic antimitotic drug), exploiting a humanized anti-FAP antibody, huB12, attached to an appropriate ADC linker and a payload. Both in vitro and in vivo experiments demonstrated an augmented efflux of proinflammatory cytokines when targeting CAFs with huB12-MMAE. The secretion of the proinflammatory cytokines interleukin (IL)-6 and IL-8 by CAFs could reprogram the immune system and orient it towards targeting malignant cells [5]. Furthermore, this FAP-targeting ADC could become a promising combinational strategy alongside already established immunotherapy options to enhance their antitumor potential [6]. OMTX705 is another ADC formed by a different humanized antibody targeting FAP, OMTX005, that has undergone preclinical evaluation. It showed satisfying antitumor action in pancreatic, lung, breast, and ovarian patient-derived xenograft (PDX) models as a monotherapy agent and in combination with other anticancer agents. It is noteworthy that the antitumor responses were durable, with no testimony of drug resistance development even after drug discontinuation and subsequent reintroduction [7]. These encouraging results prompted the initiation of a phase I clinical study of OMTX705 (NCT05547321), both as a single-agent therapy or in conjunction with the immune checkpoint inhibitor (ICI) pembrolizumab, as a salvage treatment option for patients with advanced or metastatic cancer in whom there is no approved standard of care medical intervention. This phase I trial is currently ongoing, recruiting patients with different solid malignancies.

Another appealing approach is to target signaling molecules that mediate the tumorigenic functions of CAFs. Accordingly, a phase I clinical study of LY3076226, an ADC targeting fibroblast growth factor receptor (FGFR)-3 in patients with advanced or metastatic tumors, was designed (NCT02529553) [8]. Activated CAFs secrete fibroblast growth factors (FGFs) that, in turn, potentiate FGFRs and arbitrate tumor proliferation and resistance to therapies [9]. The phase I trial demonstrated an adequate safety profile for LY3076226; however, it was terminated due to pipeline prioritization, and consequently, antitumor effects were unobtainable [8]. A different phase I clinical trial (NCT02565758) evaluated the effects of targeting the cell membrane protein leucine-rich repeat containing 15 (LRRC15) with an ADC [10]. LRRC15 is highly expressed on the surface of CAFs across various malignancies, while it is uniquely expressed on the tumor cells in different types of sarcomas [11]. ABBV-085 is the ADC assessed in the trial, comprising a humanized antibody targeting LRRC15 (PR-1498487), linked to MMAE. The safety results fulfilled the criteria, while antitumor efficacy was established primarily in patients with undifferentiated pleomorphic sarcoma and osteosarcoma [10], verifying a heightened likelihood of clinical benefit in patients with sarcomas.

Preclinical data on CAF-targeting ADCs are also encouraging. Recently, researchers investigated the combination of focal adhesion kinase (FAK) signaling targeting and either a human epidermal growth factor receptor 2 (HER2)-targeted ADC or a trophoblast antigen 2 (TROP2)-targeted ADC [12]. The rationale behind FAK signaling targeting lies in its implication in tumor progression [13], alongside its involvement in the oncogenic function of CAFs [14]. Combining the small-molecule inhibitor of FAK IN10018 with ADCs targeting either HER2 or TROP2 that have already displayed clinical benefit in various malignancies, such as breast [15] and gastric cancer [16], could enhance the efficacy of the aforementioned ADCs and extend their therapeutic profile across different solid tumors. Indeed, this dual targeting attenuated the function of CAFs, facilitating the improved uptake of the ADC into the tumor cell and augmenting the antineoplastic outcomes [12]. A different and interesting perspective is to target glypicans, proteoglycans that are abnormally expressed in multiple solid tumors [17]. Glypican-1 (GPC1) has been found to be expressed in pancreatic ductal adenocarcinoma (PDAC), both in malignant cells and CAFs, with the greatest expression of GPC1 detected in CAFs [18]. Exploiting this observation, a GPC1-targeted ADC with MMAE was preclinically developed and managed to overcome resistance to conventional anticancer drugs conferred by CAFs [18].

Overall, current anticancer approaches focus mainly on the rapidly dividing tumor cells and ignore that tumor cells thrive in an intricate milieu of divergent cells. A more holistic approach is anticipated; ADCs targeting CAFs and their integration into the already existing therapeutic modalities appears to be auspicious. However, challenges exist that cannot be overlooked. First, CAFs can either be characterized as pro-tumorigenic or anti-tumorigenic, depending on their molecular profile [19]. Targeting CAFs in general might halt their antineoplastic role, thus aiding cancer progression. Delving deeper into the molecular profile of CAFs and their phenotypic characterization is imperative to delicately target the subtypes that promote rather than hamper tumor growth. Additionally, since a universal marker that can molecularly characterize CAFs has not yet been established, it is difficult to develop an ADC that can solely and specifically target CAFs. CAFs are a highly heterogenous population, which makes this effort even more laborious [20].

As far as new perspectives in the field of CAF-targeting ADCs are concerned, targeting the urokinase plasminogen activator receptor-associated protein (uPARAP; also known as Endo180, encoded by the *MRC2* gene), a key CAF collagen receptor predominantly restricted to CAFs with little or no expression by the tumor cells, is appealing [21,22]. This receptor is expressed in both primary and metastatic sites of tumors of mesenchymal origin. Preclinical data are promising so far, both in vitro and in vivo, with minimal adverse events [23]. Innovative ADC treatment options for cancers with a high unmet medical need are currently available and continue to develop. In conclusion, CAF-targeting ADCs are novel and favorable therapeutic modalities that are nevertheless currently at an early stage of development. Future studies, both preclinical and clinical, will elucidate their precise anticancer potential and integrate them into clinical practice.
